# Anti-breast cancer effects of phytochemicals: primary, secondary, and tertiary care

**DOI:** 10.1007/s13167-022-00277-2

**Published:** 2022-04-14

**Authors:** Alena Mazurakova, Lenka Koklesova, Marek Samec, Erik Kudela, Karol Kajo, Veronika Skuciova, Sandra Hurta Csizmár, Veronika Mestanova, Martin Pec, Marian Adamkov, Raghad Khalid Al-Ishaq, Karel Smejkal, Frank A. Giordano, Dietrich Büsselberg, Kamil Biringer, Olga Golubnitschaja, Peter Kubatka

**Affiliations:** 1 Clinic of Obstetrics and Gynecology, Jessenius Faculty of Medicine, Comenius University in Bratislava, 036 01 Martin, Slovakia; 2 Department of Pathological Physiology, Jessenius Faculty of Medicine, Comenius University in Bratislava, 036 01 Martin, Slovakia; 3 Department of Pathology, St. Elizabeth Cancer Institute Hospital, 81250 Bratislava, Slovakia; 4 Unilabs Slovensko, S. R. O., Pathology, Namestie L. Svobodu 1, 974 01 Banska Bystrica , Slovakia; 5 Department of Histology and Embryology, Jessenius Faculty of Medicine, Comenius University in Bratislava, 036 01 Martin, Slovakia; 6 Department of Medical Biology, Jessenius Faculty of Medicine, Comenius University in Bratislava, 036 01 Martin, Slovakia; 7 Department of Physiology and Biophysics, Weill Cornell Medicine-Qatar, Qatar Foundation, Education City, Doha, Qatar; 8 Department of Natural Drugs, Faculty of Pharmacy, Masaryk University, Palackého třída 1946/1, 61200 Brno, Czech Republic; 9 Department of Radiation Oncology, University Hospital Bonn, Rheinische Friedrich-Wilhelms-Universität Bonn, Bonn, Germany; 10 Predictive, Preventive and Personalised (3P) Medicine, Department of Radiation Oncology, University Hospital Bonn, Rheinische Friedrich-Wilhelms-Universität Bonn, 53127 Bonn, Germany

**Keywords:** Breast cancer, Phytochemicals, Evidence-based research data, Individualized patient profiling, Modifiable risk factors, Health risk assessment, Molecular patterns, Predictive Preventive Personalized Medicine (PPPM/3PM), Primary secondary tertiary care, Treated cancer, Stroke, COVID-19, Translational research, Plants, Food, Cost-effective disease management, Improved individual outcomes

## Abstract

Breast cancer incidence is actually the highest one among all cancers. Overall breast cancer management is associated with challenges considering risk assessment and predictive diagnostics, targeted prevention of metastatic disease, appropriate treatment options, and cost-effectiveness of approaches applied. Accumulated research evidence indicates promising anti-cancer effects of phytochemicals protecting cells against malignant transformation, inhibiting carcinogenesis and metastatic spread, supporting immune system and increasing effectiveness of conventional anti-cancer therapies, among others. Molecular and sub-/cellular mechanisms are highly complex affecting several pathways considered potent targets for advanced diagnostics and cost-effective treatments. Demonstrated anti-cancer affects, therefore, are clinically relevant for improving individual outcomes and might be applicable to the primary (protection against initial cancer development), secondary (protection against potential metastatic disease development), and tertiary (towards cascading complications) care. However, a detailed data analysis is essential to adapt treatment algorithms to individuals’ and patients’ needs. Consequently, advanced concepts of patient stratification, predictive diagnostics, targeted prevention, and treatments tailored to the individualized patient profile are instrumental for the cost-effective application of natural anti-cancer substances to improve overall breast cancer management benefiting affected individuals and the society at large.

## Why 3PM concepts are pivotal to advance primary, secondary, and tertiary care in the overall breast cancer management?

According to the World Health Organization, female *breast cancer* has now surpassed lung cancer as the leading cause of *global* cancer *incidence*, as of 2021, accounting for estimated 2.3 million new annual cases (12%) worldwide [1]. Alarming statistics are particularly dramatic for the triple-negative breast cancer (TNBC): more than 50% of affected individuals in this patient cohort die within the first 6 months of the metastatic disease [2]. Patient stratification demonstrates that lymph node-positive TNBC patients being slim (BMI < 18.5) have particularly poor outcomes [3]. Young patients aged below 35 years diagnosed with metastatic breast cancer demonstrate significantly lower 5-year disease-free survival and significantly higher prevalence of distant metastasis compared to their counterparts aged above ≥ 65 years [4]. Rapidly increasing breast cancer incidence in general and, in particular, aggressive metastatic breast cancer sub-types in young female populations prompt application of advanced screening programmes, and targeted preventive and individualized approaches in overall breast cancer management [5]. To this end, primary (sub-optimal health conditions with evident risks of the disease predisposition), secondary (predisposition to the metastatic breast cancer), and tertiary (making palliative care to the management of chronic disease) care in the framework of predictive, preventive, and personalized medicine is considered an advanced strategy to improve individual outcomes and cost-efficacy of the disease management [6–10].

## Phytochemicals are instrumental to advance cancer management

**Fig. 1:**
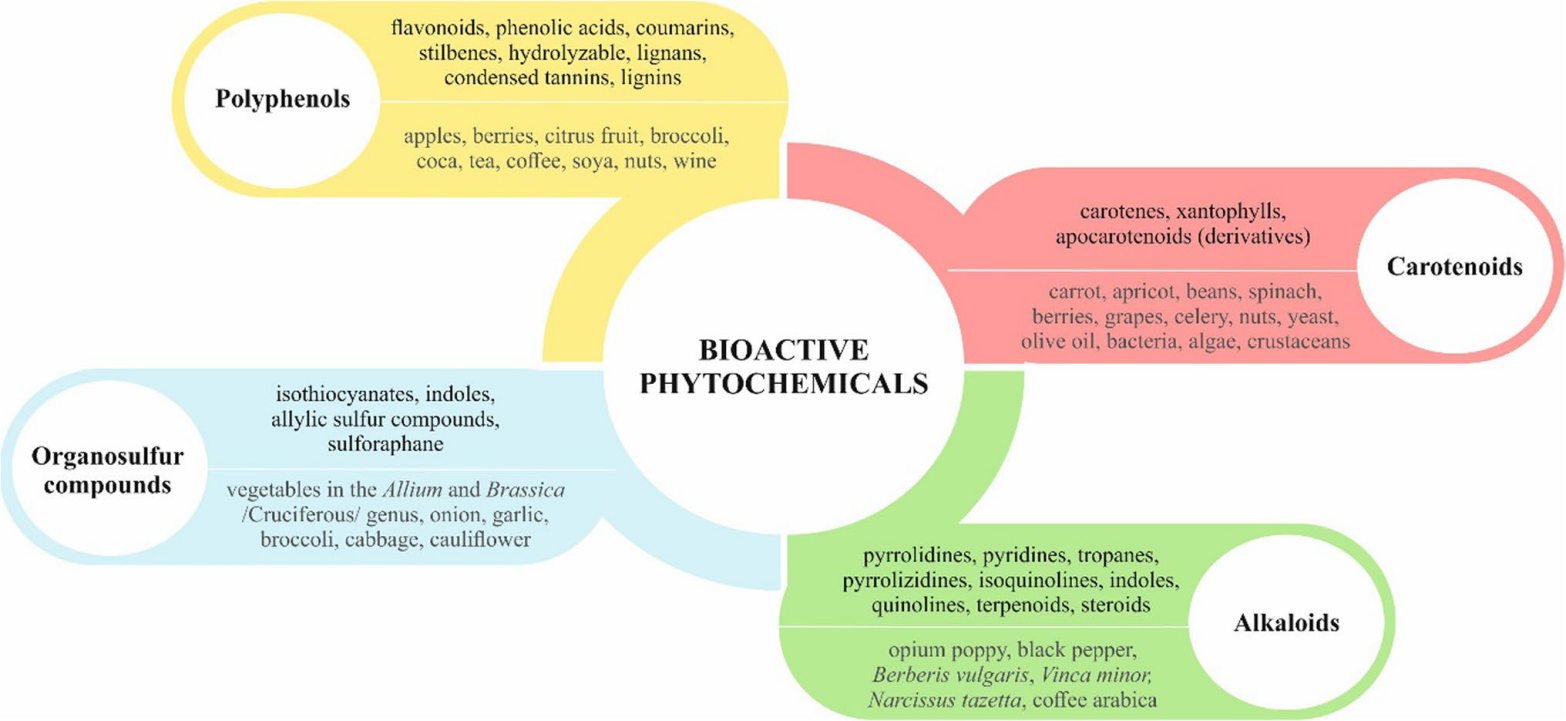
**Classification and main sources of phytochemicals** [12–17]. Polyphenols, carotenoids, organosulfur compounds, and alkaloids represent examples of large classes of phytochemicals. Polyphenols (plant phenolics) are further divided into flavonoids, phenolic acids, coumarins, stilbenes, hydrolyzable, condensed tannins, lignans, and lignins [13]. Indeed, current evidence state over 8000 identified polyphenols, but the number is largely underestimated [18]. Carotenoids are naturally occurring liposoluble pigments (red, orange, yellow) of fruits and vegetables and can be found also in fungi, algae, and bacteria. Nearly 700 carotenoids have been identified and can be further divided into carotenes, xantophylls, and apocarotenoids (derivatives) [14]. The presence of nitrogen atoms/s is a basic characteristic of alkaloids [19]. Alkaloids can be subdivided into numerous sub-classes. As stated by Heinrich et al. in October 2020, the *Dictionary of Natural Products* included 27,683 alkaloids [20]. Organosulfur compounds include isothiocyanates, indoles, allylic sulfur compounds, and sulforaphane [15]

Phytochemicals as secondary plant metabolites represent non-nutrient chemical compounds of plants [11]. The sources of phytochemicals include vegetables, fruits, grains, beverages, and medicinal plants [12]. Examples of bioactive phytochemicals are represented by numerous polyphenols, carotenoids, organosulfur compounds, and alkaloids (Fig. 1) [13].

Traditional Chinese medicine (TCM) herbs has been used to prevent and treat diseases for thousands of years [21]. It is currently investigated whether it could prevent or treat cancer or enhance conventional anticancer therapies [22]. Written records of plants used for medicinal purposes date back 5000 years to Sumerians, but archaeological studies provided evidence of using medicinal plants 60,000 years ago in an area now called Iraq. Despite the long history of use and many times effectivity, the use of herbal medicine declined with the progress of modern or conventional Western medicine due to the lack of scientific proofs and verifications [23]. Nevertheless, recent research and the modern approach of the individual persons to their health status highlight the potential of phytochemicals for human health. Phytochemicals, either isolated or their mixtures in whole plant foods, exert many biological activities, including antioxidant, immunomodulatory, anti-microbial, anti-inflammatory, and anticancer. In addition, phytochemicals might have only minimal side effects, are cost-effective, and are usually widely available. Nevertheless, the usability of phytochemicals faces several complications, such as a low bioavailability due to their rapid and extensive metabolism, which is commonly individual in patient and for each phytochemical different [12, 24–27]. But these negatives do not disclassify phytochemicals and their use as a traditional or modern treatment of various diseases [28]. Preclinical research highlights the vast potential of phytochemical to combat breast cancer functioning as preventive or therapeutic agents [24, 29–31].

## Pleiotropic effects of phytochemicals demonstrated in vitro and in vivo

Recent evidence demonstrates pleiotropic effects of phytochemicals (isolated or their mixtures) on multiple signalling pathways and suppresses malignant transformation in vitro and in vivo [32].

Results of experiments showed a direct association between the application of phytochemicals and the suppression of cancer development through the modulation of different cell signalling pathways [33]. Numerous phytoconstituents in function food regulate events connected with malignant transformation, such as apoptosis [26], proliferation [34], angiogenesis [27], inflammation [35], invasiveness, and metastasis [12]. Many studies described the significant role of phytochemicals in modulating epigenetic changes and metabolic reprogramming—key events for cancer initiation, promotion, and progression [36, 37]. Preclinical trials using breast cancer models demonstrated oncostatic effects of secondary metabolites of plants via modulation of critical enzymes contributing to aerobic glycolysis [38], detoxification and antioxidant machinery [39], DNA repair mechanisms [40], or programmed cell death [25]. In addition, phytochemicals can in experimental models of breast cancer restore global and gene-specific promoter DNA methylation patterns [41], alter the gene expression by modulating miRNA expression [42], or affect histone modifications [43].

Multiple chemopreventive and oncostatic effects of phytochemicals observed in preclinical research represent a promising avenue in cancer management, which could be introduced in the clinical sphere. More in-depth investigations of the role of phytochemicals will accelerate the development of novel therapeutic strategies to improve life quality and the overall survival of patients with breast cancer.

## Current state of clinical evidence on the effectiveness of phytochemicals in breast cancer management

Despite intensive evaluation in preclinical cancer research, limited evidence is available for the effectiveness of natural compounds in the clinical sphere [27]. Nevertheless, current clinical studies provide valuable evidence on effectiveness of phytochemicals in breast cancer management, especially of breast cancer preventive agents, phytochemicals as therapeutic agents, phytochemicals combined with conventional therapies (e.g. chemotherapy), or phytochemicals mitigating the side effects of current therapeutic approaches.

### Phytochemicals in breast cancer prevention

The biological activities of fruits, vegetables, or medicinal plants are attributed to the presence of phytochemicals (isolated or their mixtures) [12, 25, 44, 45]. Despite the crucial anticancer activity of isolated phytochemicals, current research highlights the potent additive or synergic effects of mixtures of phytochemicals in whole plant food [24, 27, 29, 44, 46–48]. Healthy diet patterns characterized by a greater intake of whole plant-based food have been on a long-term basis associated with a reduced risk of cancer. Previous studies showed a reduced risk of breast cancer associated with a Mediterranean diet [49, 50], with a consumption of a specific type of fruits (peaches/nectarines) [51], or with polyphenols present in green tea [52].

Table 1 shows the current clinical evaluations conducted on breast cancer patients and analyses the association between breast cancer risk and dietary patterns.

**Table 1:** Current clinical studies evaluating the effects of phytochemicals in breast cancer prevention

Phytochemical	Study design	Year	Study participants (number)	Results	Reference
dTAC	Hospital-based case–control study	2021	Women with pathologically confirmed BC (*n* = 412) and healthy controls (*n* = 456)	Inverse association between dTAC and odds of BC (whole population); highest quartile of dTAC → 0.39 times less probability of BC vs. the lowest quartile; postmenopausal women with greatest dTAC had lower odds for BC vs. those with the lowest dTAC	[53]
Legumes and nuts	Population-based case–control study	2021	New BC cases (*n* = 350) and controls (*n* = 700)	Inverse association between the consumption of legumes and nuts and odds of BC	[54]
Plant-based diet	Population-based case–control study	2021	Newly diagnosed BC (*n* = 350) and healthy controls (*n* = 700)	Inverse association between adherence to plant-based diet index and hypothesized healthy plant-based diet index with breast cancer risk	[55]
HEI	Case–control study	2020	BC cases (*n* = 134) and cancer-free controls (*n* = 265)	HEI associated with decreased BC risk	[56]
PVG	Prospective cohort study	2020	Women in the Seguimiento Universidad de Navarra cohort (*n* = 10,812)	Inverse association with BC for a modest overall PVG, but not for hPVG and uPVG separately	[57]
Fruits and vegetables	Data from prospective cohort study – NHS	2019	NHS (*n* = 88,301) and NHSII (*n* = 93,844)	Fruits and vegetables (especially cruciferous and yellow or orange vegetables) associated with lower risk of BC, total vegetables with lower risk of oestrogen receptor negative BC, total fruits and vegetables with lower risk of HER2-enriched, basal-like, and luminal A BC	[58]
24 months of dietary or physical intervention	Randomized intervention trial	2019	Healthy postmenopausal women (*n* = 226)	Reduced mammographic breast density	[59]
Healthier diet	Case–control study	2018	BC cases (*n* = 145) and controls (*n* = 148)	Healthier diet (vitamin A, β-carotene, vitamin C, and folate) associated with decreased risk of BC vs. high fat and lamb meat	[60]
Bean fiber, beans, grains	Food frequency data from population-based case–control study	2018	BC cases (*n* = 2135) and controls (*n* = 2571)	Greater intake of bean fiber, beans, grains may lower the risk of ER- and PR- BC	[61]
Cruciferous vegetables, glucosinolates, and isothiocyanate	Case–control study	2018	BC cases (*n* = 1485) and controls (*n* = 1506)	Indicated association between higher intake of cruciferous vegetable, glucosinolates, and isothiocyanates and BC risk in Chinese women	[62]
Green tea extract	Randomized, double-blinded, placebo-controlled phase II clinical trial	2017	Healthy postmenopausal women (*n* = 1075)	Reduced PMD in younger women when compared with placebo group	[63]
MD	Multivariate case–control analyses	2017	Incident BC cases (*n* = 2321) and subcohort members (*n* = 1665)	Moderately strong inverse associations with risk of ER- (40% reduction), and ER- PR- (39% reduction) breast cancers	[64]

*BC*; breast cancer, *dTAC*; dietary total antioxidant capacity, *ER-*; oestrogen receptor negative, *HEI*; Healthy Eating Index, *HER2*; human epidermal growth factors receptor 2, *hPVG*; healthful pro-vegetarian dietary pattern, *MD*; Mediterranean diet, *NHS*; Nurses’ Health Study, *PMD*; percent mammographic density, *PR-*; progesterone receptor negative, *PVG;* pro-vegetarian dietary pattern, *uPVG*; unhealthful pro-vegetarian dietary pattern

A recent case–control study in a Middle Eastern country demonstrated an inverse association (*P* < 0.05) between dietary total antioxidant capacity and the risk of breast cancer [53]. Similarly, an inverse association between legumes and nuts as the whole food group and breast cancer was observed in Iranian women [54]. Also, a population-based case–control study in Iranian women demonstrated an inverse association between adherence to plant-based diet index and hypothesized healthy plant-based diet index connected with breast cancer risk [55]. The Healthy Eating Index (HEI) is a standard measure of diet quality that assesses the quality of diet based on the evaluation of the intake of total fruits, whole fruits, total vegetables, greens and beans, whole grains, dairy, total food proteins, seafood and plant proteins, fatty acids, refined grains, sodium, added sugars, and saturated fats. Indeed, the HEI score is associated with a decreased risk of breast cancer [56]. Moreover, pro-vegetarian dietary patterns are predominantly plant-based or plant-forward but not fully vegetarian or vegan. A large prospective study conducted on a Mediterranean cohort described that a moderate adherence to pro-vegetarian dietary patterns might decrease the risk of breast cancer [57]. Interestingly, Farvid et al. (2019) evaluated a plant-based diet and the risk of breast cancer through repeated measures over 30 years. The results showed the association of larger fruit and vegetable intake, especially cruciferous and yellow or orange vegetables, and a lower risk of breast cancer. The intake of total vegetables was linked to a lower risk of oestrogen receptor-negative (ER-) breast cancer, higher intake of total fruits and vegetables with a lower risk of HER2-enriched, basal-like, and luminal A breast cancer molecular subtypes [58]. Similarly, women consuming a healthier diet (β-carotene, vitamins A and C, and folate) were at decreased risk of breast cancer compared with women with a diet characterized by high fat and lamb meat [60]. In addition, greater intake of bean fiber, beans, and grains may lower the risk of ER- and progesterone receptor-negative (PR-) breast cancer [61]. Moreover, a higher intake of cruciferous vegetable, glucosinolates, and isothiocyanates is indicated to be inversely associated with breast cancer risk in Chinese women [62]. Mammographic density (MD) describes the relative amount of fibroglandular to fat tissue, which is often reported as percent MD (PMD)—a proportion of dense area over total breast area [63]. MD is considered an independent risk factor for breast cancer [59]. Women with ≥ 75% MD are associated with a 4 to 6 times greater risk of breast cancer than women with MD below 10% [63]. Nevertheless, a randomized intervention trial suggested that a 24-month dietary intervention (plant foods with a low glycaemic load, low in saturated fats and alcohol) or physical training (combining daily moderate-intensity activities and session of more strenuous activity once per week) could reduce mammographic breast density [59]. Similarly, green tea extract supplementation reduced PMD in younger women compared with the placebo group [63]. Multivariate case–control analyses revealed an inverse association between adherence to the Mediterranean Diet and risk of ER- postmenopausal breast cancer (approximately 40% reduction of risk), but the study did not show significant association with oestrogen receptor positive (ER +) or total breast cancer risk. These results provide proofs of special benefits of diet containing large amounts of phytochemicals in the breast cancer prevention due to the poor prognosis of ER- breast cancer subtype [64].

However, for more efficient chemopreventive analyses, surrogate endpoint biomarkers should be validated for the short-term assessment of breast cancer risk. Still, any chemopreventive agent should be precisely evaluated, for example by assessing potential long-term toxicity during repeated use [65].

### Phytochemicals mitigating side effects of cancer therapy

The benefits of utilization of phytochemicals are also associated with reducing the side effects of conventional therapeutic strategies. Table 2 provides an overview of the current clinical research conducted on the effects of phytochemicals to mitigate the side effects of conventional therapeutic approaches against breast cancer; indeed, these studies are specified in Table 2.

**Table 2:** The overview of clinical trials on phytochemicals utilized in mitigating side effects of breast cancer conventional therapies (surgery, radiotherapy, chemotherapy, and targeted therapy by aromatase inhibitors)

Phytochemical	Study design	Year	Study participants (number)	Results	Reference
***Surgery***					
Linfadren® (contains diosmin, *Mellilotus officinalis* extract rich in coumarins, and *Uva ursi* extract rich in arbutin)	Randomized controlled trial	2019	BCRL patients (*n* = 50)	Safe and effective when combined with CDT for reducing BCRL when compared with CDT alone	[66]
***Radiotherapy***					
Silymarin	Randomized, double-blind, placebo-controlled clinical trial	2019	BC patients undergoing radiotherapy (*n* = 40); silymarin or placebo group on chest wall skin following radical mastectomy and starting at the first day of radiotherapy	Reduced severity of radiodermatitis	[67]
*Nigella sativa* L. extract	Randomized, double-blind, placebo-controlled, clinical trial	2019	BC patients undergoing radiotherapy (*n* = 62); *Nigella sativa* gel group or placebo group	Decreased severity of ARD and delayed moist desquamation onset	[68]
EGCG	Single-institution phase II trial	2016	BC patients that underwent mastectomy followed by adjuvant radiotherapy (*n* = 49); topical EGCG applied daily (starting when grade I dermatitis appeared and ending 2 weeks after radiotherapy)	Reduced pain in 85.7% of patients, burning feeling in 89.8%, itching in 87.8%, pulling in 71.4%, and tenderness in 79.6%	[69]
***Chemotherapy***					
Nutritional intervention on healthy eating (emphasizing fruits, vegetables, grains, fat-free or low-fat milk products; low saturated fats, *trans* fats, cholesterol, salt, and added sugars)	Randomized controlled trial	2021	BC patients at the beginning of neoadjuvant chemotherapy (doxorubicin, cyclophosphamide, and an intravenous antiemetic pattern); intervention group receiving an individualized diet plan (*n* = 19) and control group (*n* = 15)	Preserved the role function of QoL and handgrip strength reduced the occurrence of nausea/vomiting, loss of appetite, and the frequency of leukopenia and abdominal pain	[70]
*PG* (thew form not stated)	Randomized, double-blind, placebo-controlled trial	2020	Early BC patients (*n* = 125) receiving AT-based chemotherapy, randomized into PG group or placebo group	Lower incidence of subclinical heart failure and lower cardiac troponin T levels in PG group vs placebo group	[71]
Peppermint (Super mint oral drop™)	Randomized controlled trial	2020	BC patients undergoing chemotherapy, experimental group received 40 drops of peppermint extract (*n* = 42) and control group (*n* = 42)	Mean score of severity of nausea, vomiting, and anorexia lower in experimental group vs. control group	[72]
Mistletoe extracts (Helixor A™, Iscador M Spez™)	Randomized controlled trial	2018	95 BC patients undergoing surgery and adjuvant chemotherapy with cyclophosphamide, adriamycin, and 5-fluorouracil; mistletoe extracts group (*n* = 64) injected three times/week week during 18 weeks of chemotherapy and control group (*n* = 31)	Trend toward less neutropenia and improved pain and appetite loss scores in breast cancer patients	[73]
Ginger (powdered rhizoma)	Randomized, double-blind and clinical trial study	2016	BC patients undergoing chemotherapy (*n* = 65); ginger group – regimen for 5 days before and 5 days after chemotherapy (twice daily 500-mg capsules of powdered ginger root)	Effective to relieve chemotherapy-induced nausea and vomiting (reduced vomiting, frequency of nausea)	[74]
***Aromatase inhibitors***					
Hydroxytyrosol, omega-3 fatty acids, and curcumin	Prospective, multicentre, open-label, single-arm, clinical trial	2019	Post-menopausal BC patients (*n* = 45) with elevated CRP receiving predominantly AI	Reduce pain and inflammation (decreased CRP) in breast cancer patients with aromatase-induced musculoskeletal symptoms	[75]
Yi Shen Jian Gu	Randomized controlled trial	2018	Postmenopausal BC patients (*n* = 77) with hormone-receptor positive stage I – III receiving AI	Improved musculoskeletal conditions	[76]

*AI*; aromatase inhibitors, *ARD*; acute radiation dermatitis, *AT*; anthracycline, *BC*; breast cancer, *BCRL*; breast cancer-related lymphedema, *CDT*; complex decongestive therapy, *CRP*; C-reactive protein, *EGCG*; epigallocatechin-3-gallate, *PG*; *Platycodon grandiflorum,* *QoL*; quality of life, *TCM*; traditional Chinese medicine

For decades, surgery represented a mainstay in breast cancer therapy [77]. Breast cancer-related lymphedema (BCRL), described as a regional and generalized accumulation of lymph fluid in the interstitial space of the upper limb due to the interruption of the axillary lymphatic system, is a common complication associated with breast cancer surgery. Indeed, Linfadren® that contains flavonoid diosmin, coumarins, and phenolic glycoside arbutin was safe and effective when combined with complex decongestive therapy (CDT) for reducing BCRL compared to CDT alone [66].

Except for surgery, the primary treatment modalities for breast cancer include radiation, chemotherapy, hormone-based therapies, or targeted therapy [78, 79]. Indeed, conventional breast cancer therapies are associated with various adverse effects. Topical application of gel with silymarin, a flavonoid extracted from *Silybum marianum* (silymarin gel 1% containing extract with 80% of flavonolignans including silybin, silychristin, silydianin, 2,3‐dehydrosilybin, and 2,3‐dihydrosilychristin once daily), reduced radiodermatitis’s severity and delays its occurrence after 5 weeks of application in breast cancer patients undergoing radiotherapy [67]. Similarly, *Nigela sativa* L. extract (5% gel administered twice a day at least two hours before and after radiotherapy) decreased the severity of acute radiation dermatitis and delayed moist desquamation onset in breast cancer patients undergoing radiotherapy [68]. As stated above, epidemiologic studies conclude that green teas decrease the risk of breast cancer [52]. The flavonoid epigallocatechin-3-gallate (EGCG) represents the most abundant catechin in green tea and is most likely responsible for the potent biological activity of green tea [80]. A study by Zhu et al. (2016) stated the topical EGCG as an effective treatment of dermatitis induced by radiation. They demonstrated reduced pain, burning feeling, itching, pulling, and tenderness in breast cancer patients undergoing a mastectomy followed by radiotherapy; besides, no grade 3 or 4 dermatitis was observed in patients receiving EGCG even though receiving the same dose of radiation [69].

Worsened quality of life (QoL) and toxicity is common during breast cancer therapy. For example, an impaired nutritional status due to gastro-intestinal toxicity (nausea, vomiting) during chemotherapy during prolonged time may result in the interruption of the treatment. Worsening QoL might be related to worsened prognosis and an increase in the risk of recurrence [70]. However, the nutritional intervention on healthy eating (emphasizing fruits, vegetables, grains, among others) during the first three cycles of neoadjuvant chemotherapy could minimize clinical involutions during therapy and therapy interruptions. This nutritional intervention preserved QoL and handgrip strength showing decreased occurrence of nausea/vomiting and loss of appetite, and lower frequency of leukopenia and abdominal pain when compared with the control [70]. Anthracycline-based chemotherapy is a backbone of adjuvant breast cancer therapy. However, anthracycline-based chemotherapy is associated with myelosuppression, hair loss, and cardiotoxicity as the most serious side effect. An administration of phytochemicals or herbs could represent a potent shift to prevent anthracycline-induced cardiotoxicity. *Platycodon grandiflorum* that has been long used in traditional Chinese medicine to treat cardiovascular diseases prevented acute and chronic cardiac injury induced by anthracycline in early breast cancer patients without compromising the effects of the chemotherapy. However, the antitumor activity itself was not affected [71]. Peppermint (*Mentha piperita*) extract exerted a significant decrease in the man score of severity of nausea, vomiting, and anorexia in breast cancer patients undergoing chemotherapy compared with the control group [72]. The subcutaneous injection of mistletoe extracts showed a trend toward less neutropenia and improved pain and appetite loss scores in breast cancer patients undergoing surgery and adjuvant chemotherapy with cyclophosphamide, adriamycin, and 5-fluorouracil. However, the mistletoe extracts did not affect the frequency of relapse or metastasis within 5 years [73]. Also, ginger capsules relieved chemotherapy-induced nausea and vomiting in breast cancer patients undergoing chemotherapy [74].

Oestrogens are crucially involved in the pathogenesis of breast cancer. Aromatase catalyses essential steps in the biosynthesis of oestrogen; and thus, aromatase inhibitors represent effective targeted therapy in ER + breast cancer patients [81]. Musculoskeletal symptoms (bone pain, arthralgia, myalgia, carpal tunnel syndrome, trigger finger) develop in approximately half of the patients on aromatase inhibitors. Therefore, aromatase inhibitor-associated musculoskeletal symptoms (AIMSS) among patients on aromatase inhibitors result in lower QoL and poor adherence to aromatase inhibitors. Indeed, the discontinuation of therapy due to musculoskeletal symptoms occurs in up to 20% of them. Indeed, early discontinuation and non-adherence to aromatase inhibitors are related to an increase in mortality of breast cancer patients [76]. The conventional therapy for AIMSS includes NSAIDs, analgesics, or vitamin D. Still, there is a need for more effective AIMSS management [76]. Therefore, a pilot study by Martínez et al. (2019) demonstrated that olive-derived polyphenol hydroxytyrosol combined with omega-3 fatty acids and curcumin could reduce pain and inflammation (indicated by decreased CRP) in breast cancer patients with aromatase-induced musculoskeletal symptoms [75].

Improved musculoskeletal symptoms were observed in patients with AIMSS after an administration of preparation from Chinese medicine (Yi Shen Jian Gu granules composed of twelve herbs including *Caulis trachelospermi* (LuoShiTeng), *Fructus corni* (ShanZhuYu), *Phryma leptostachya* (TouGuCao), *Poria* (FuLing), *Radix achyranthis bidentatae* (NiuXi), *Radix angelicae sinensis* (DangGui), *Radix paeoniae albae* (BaiShao), *Radix rehmanniae preparatae* (ShuDiHuang), *Rhizoma chuanxiong* (ChuanXiong), *Rhizoma corydalis* (YanHuSuo), *Rhizoma cyperi* (XiangFu), and *Semen cuscutae* (TuSiZi)) [76].

In addition to the side effects of above discussed anticancer strategies, depression, anxiety, and psychosocial distress can occur in up to half of the patients after breast cancer diagnosis and mastectomy. Also, recently emphasized enhanced recovery after surgery (ERAS) protocols highlight the reduction of opioid analgesics and anxiolytics in patients undergoing microvascular breast reconstruction to reduce risk factors such as an overdose or respiratory distress and reduce comorbid psychological symptoms such as depression and anxiety. Shammas et al. (2021) evaluated effects of lavender oil but observed no advantages in treatment of depression, anxiety, sleep, or pain of patients undergoing microvascular breast reconstruction [82]. On the contrary, lavender oil inhaled before breast surgery decreased anxiety in breast cancer patients [83]. Therefore, low-risk therapeutics of natural origin lowering pain, anxiety, and depression still wait to be identified.

In summary, phytochemicals and their mixtures in whole plants exert potent capacity to combat the side effects of current anticancer conventional therapeutic strategies. However, the clinical research in this area can still be considered poor and requires a comprehensive approach that would allow broad use of phytochemicals in clinical practice.

### Phytochemicals combined with conventional anticancer agents

Conventional anticancer strategies combined with phytochemicals are a promising strategy in the treatment of breast cancer. An early performed clinical trial (2012) conducted on ten breast cancer patients undergoing radiotherapy demonstrated the potential of EGCG to enhance radiotherapy demonstrated through decreased levels of vascular endothelial growth factor (VEGF), hepatocyte growth factor (HGF), and reduced activation of matrix metalloproteinase (MMP)-9 and MMP-2 in patients receiving radiotherapy plus EGCG compared to patients receiving radiotherapy alone. Therefore, these results supported the potential of EGCG to serve as an adjuvant against metastatic breast cancer [84]. Similarly, a double-blinded, randomized controlled clinical trial (2015) conducted on fifty-six breast cancer patients revealed that regular onion administration was associated with reduced tumour biomarkers carcinoembryonic antigen (CEA) and cancer antigen 125 (CA-125) during doxorubicin chemotherapy [85].

In addition, as shown in Table 3, also recent breast cancer clinical evaluations provided promising results on the efficacy of phytochemicals enhancing the anticancer effect of conventional therapeutics.

**Table 3:** Phytochemicals enhancing conventional anticancer strategies (current state of clinical evidence)

Phytochemical	Study design	Year	Study participants (number)	Results	Reference
Chinese medicine	Pilot randomized controlled trial	2021	Patients randomized to receive chemotherapy plus Chinese medicine or chemotherapy alone	Enhanced effectiveness (lower CEA, CA 125, CA 153) and mitigated side effects (cardiac events, less significant reduction of white blood cells, better hepatic function) of trastuzumab-containing chemotherapy	[86]
FMD	Multicentre randomized phase 2 DIRECT trial	2020	Patients with neoadjuvant chemotherapy for HER2-negative stage II/III (*n* = 131)	More likely occurring Miller and Payne 4/5 pathological response that indicates 90–100% tumour-cell loss, and reduced DNA damage in lymphocytes induced by chemotherapy	[87]
Curcumin combined with paclitaxel	Comparative, randomized, double-blind placebo-controlled clinical trial	2020	Advanced, metastatic breast cancer patients (*n* = 150)	Curcumin and paclitaxel combination demonstrated to be superior to paclitaxel-placebo group (ORR and physical performance); curcumin demonstrated no safety issues, no reduction of QoL; curcumin could be also an effective agent to reduce fatigue	[88]
Combination therapy including chemotherapeutic regimens and arglabin	Randomized controlled trial	2018	LABC patients (*n* = 93)—experimental and control groups	Arglabin included in AC regimen resulted in an increase in 3-year disease-free survival by 28% when compared with standard regimen	[89]
Fresh yellow onion	Parallel-design, randomized, triple-blind, controlled clinical trial	2017	BC patients (*n* = 56) diagnosed with invasive ductal carcinoma following the second cycle of doxorubicin-based chemotherapy	Ameliorated insulin resistance and hyperglycaemia	[90]

*BC*; breast cancer, *CA-125*, cancer antigen 125; *CA-153*, cancer antigen 153; *CEA*, carcinoembryonic antigen; *DNA*, deoxyribonucleic acid; FMD, fasting mimicking diet; *HER2*, human epidermal growth factors receptor 2; *LABC*, locally advanced breast cancer; *ORR*, objective response rate; *QoL*, quality of life

A recent randomized controlled pilot trial by Xu et al. (2021) evaluated the effects of traditional Chinese medicine (based on mixing *Poria cocos* (Fu Ling), *Atractylodes macrocephala* (Bai Zhu), *Ginger pinellia* (Jiang Ban Xia), *Fritillaria thunbergii* (Zhe Bei Mu), *Curcuma aromatica* (Yu Jin), *Scutellaria baicalensis* (Huang Qin), *Curcuma zedoaria* (E Zhu), *Citrus aurantium* (Zhi Qiao), *Citrus reticulata* (Chen Pi), *Solanum lyratum* (Bai Ying), chicken’s gizzard-membrane (Ji Nei Jin), turtle carapace *Trionyx sinensis* (Bie Jia), *Glycyrrhiza glabra* (Gan Cao), and centipede *Scolopendra subspinipes* (Wu Gong)) in patients receiving chemotherapy for HER2-positive breast cancer. The authors concluded that personalization of traditional Chinese medicine could enhance effectiveness (lower predictive markers CEA, CA 125, CA 153) and mitigate side effects (cardiac events, less significant reduction of white blood cells, better hepatic function) of trastuzumab-containing chemotherapy in HER2-positive breast cancer patients [86]. Moreover, fasting-mimicking diet (FMD) based on a 4-day plant-based low amino-acid substitution food as an adjunct to neoadjuvant chemotherapy for HER2-negative stage II/III resulted in more likely occurring Miller and Payne 4/5 pathological response that indicates 90–100% tumour-cell loss and reduced DNA damage in lymphocytes induced by chemotherapy [87]. Furthermore, a recent comparative, randomized, double-blinded placebo-controlled clinical trial by Saghatelyan (2020) evaluated the efficacy and safety of curcumin combined with paclitaxel in advanced, metastatic breast cancer patients. The combination of curcumin and paclitaxel was superior to a paclitaxel-placebo group in terms of objective response rate (ORR) and physical performance. Also, curcumin demonstrated no safety issues and no reduction of QoL, and could also be an effective agent to reduce fatigue [88]. Arglabin is a sesquiterpene guaianolide γ-lactone isolated from *Artemisia glabella* [91], an endemic plant of Central Kazakhstan. Long-term results of combination therapy of locally advanced breast cancer including chemotherapeutic regimens and arglabin revealed that arglabin included in AC regimen increases 3-year disease-free survival by 28% when compared with the standard regimen [89]. Fresh yellow onion ameliorated insulin resistance and hyperglycaemia in breast cancer patients during doxorubicin-based chemotherapy. Thus, a high onion intake represents possible promising synergistic effect in doxorubicin-based chemotherapy [90].

In 2017, Meng et al. presented a prospective cohort study protocol to evaluate TCM herbs to treat triple-negative breast cancer described by aggressive behaviour, rapid progression, low disease-free survival, and high risk of recurrence and metastasis. The association between CHM and recurrence and metastasis rate represents the study’s primary objective. In contrast, secondary objectives include the relationship between QoL and CHM and the correlation between symptoms (sleep quality, fatigue, depression, anxiety) related to breast cancer and CHM [22]. The results of the study are to be evaluated.

In summary, recent evidence supports the potential of phytochemicals in combination with conventional anticancer agents. However, expanded comprehensive analyses of the effects of phytochemicals enhancing conventional therapy are necessary to advance breast cancer management and identify better therapeutic strategies.

### Phytochemicals in breast cancer treatment

Previously published studies indicate the efficacy of naturally occurring phytochemicals (not combined with conventional anticancer therapies) in the treatment of breast cancer. In a phase IB (2012) [65] and IIB (2015) dose-escalation trial, Crew et al. highlighted the efficacy of polyphenon E, a green tea extract, to affect biomarkers of tumour growth signalling and angiogenesis in hormone-receptor negative breast cancer. The extract specifically decreased serum HGF involved in tumour growth, migration, invasion, and VEGF, essential for tumour angiogenesis [92]. Some other early clinical studies also indicated potent anticancer effects of phytochemicals, based on 12-month dietary changes such as vegetables or fruits among others (2015). The dietary changes positively changed LUMA DNA and LINE-1 DNA methylation [93]. A phenolic compound *trans*-resveratrol (2012) decreased methylation of tumour suppressor RASSF-1α [94]. In addition, a phase IB dose-escalation study by Perez et al. (2010) demonstrated that *Scutellaria barbata* (BZL101) is safe and well-tolerated and exerts promising anticancer potential in women with metastatic breast cancer [95].

In addition to earlier dated studies, more recent clinical evaluations (Table 4) evaluated the effects of phytochemicals (not combined with conventional anticancer therapies) in breast cancer therapy.

**Table 4:** Overview of current clinical evaluation of the effect of phytochemicals in breast cancer therapy (not combined with conventional anticancer therapies)

Phytochemical	Study design	Year	Study participants (number)	Results	Reference
Low-fat dietary pattern (fruits, vegetables, grains)	Long-term follow-up of the Women’s Health Initiative Randomized Trial	2020	Postmenopausal women (*n* = 48,835)	Reduced risk of death in post-menopausal breast cancer patients	[96]
CHM	Clinical trial and network pharmacology	2017	Metastatic breast cancer patients (*n* = 182)	Correlation with favourable survival outcomes (potentially through inhibition of HSP90, ERα, and TOP-II related pathways)	[97]
GSP		2017	Early breast cancer patients (*n* = 12) receiving GSP 300 mg, equivalent to 44.9 mg of EGCG, daily for 4 weeks prior to surgery	Total EGCG detectable in all tumour tissue; median total EGCG concentration higher in tumour vs. adjacent normal tissue; free EGCG plasma levels positive correlation with the Ki-67 decrease in tumour	[98]
Orally taken silybin-phosphatidylcholine	Clinical trial	2016	BC patients (*n* = 12)	High silybin blood concentration and selectively accumulation in breast tumour tissue	[99]
Long-term pre-diagnosis consumption of soy	Population-based cohort study	2016	TNBC patients (*n* = 272)	Over-expressed microRNA-29a-3p and *IGF1R* and under-expressed *KRAS* and *FGFR4*	[100]
Cruciferous vegetables	Clinical trial baseline	2016	Women with abnormal mammogram findings scheduled for breast biopsy (*n* = 54)	Intake of cruciferous vegetables associated with decreased cell proliferation (decreased Ki67 in DCIS but not in benign tissues or IDC)	[101]
Short-term dietary intervention on nutrition education	Randomized controlled trial	2016	Spanish-speaking women (*n* = 70) with a history of stage 0–III breast cancer	Efficacy in increasing vegetables and fruits intake and altered biomarker of BC recurrence risk—increase in global DNA methylation	[102]

*BC*; breast cancer, *CDT*; complex decongestive therapy, *CHM*; Chinese herbal medicine, *DCIS*; ductal carcinoma in situ, *DNA*; deoxyribonucleic acid, *EGCG*; epigallocatechin-3-O-gallate, *ERα*; oestrogen receptor alpha, *GSP*; Greenselect Phytosome, *HSP90*; heat shock protein90, *IDC*; invasive ductal carcinoma, *TNBC*; triple-negative breast cancer, *TOP-II*; topoisomerase II

Long-term follow-up of the Women’s Health Initiative Randomized Trial recently highlighted that low-fat dietary patterns characterized by increased fruits, vegetables, and grains might reduce the risk of death in postmenopausal breast cancer patients [96]. Moreover, Lazzeroni et al. (2017) conducted a presurgical study to evaluate the effects of lecithin formulation of green tea extract (Greenselect Phytosome®, GSP) in early breast cancer patients. Total EGCG content was analysed in all tumour tissues and the concentration was found to be higher than that in normal tissue. Moreover, free EGCG plasma level correlated with decreased Ki67, a marker of tumour cell proliferation, in tumour tissue. Therefore, these results highlight the increase of EGCG bioavailability through oral GSP and its possible antiproliferative effects in breast cancer patients [98]. Similarly, a year earlier, Lazzeroni et al. (2016) demonstrated that silybin-phosphatidylcholine administration (orally bioavailable complex of a flavonoid silybin) resulted in high silybin blood concentration and selective accumulation in breast tumour tissue of early breast cancer patients. These results can serve as a basis for future clinical evaluation of silybin in breast cancer prevention [99]. Moreover, long-term pre-diagnosis consumption of soy may result in an increased expression of tumour suppressor microRNA and genes and decreased expression of oncogenes as demonstrated by over-expressed microRNA-29a-3p and *IGF1R* and under-expressed *KRAS* and *FGFR4* in TNBC tissues of women with high soy intake [100]. Moreover, the intake of cruciferous vegetables was associated with a decrease in cell proliferation demonstrated by lower protein expression of Ki67 in ductal carcinoma in situ but not in benign or invasive ductal carcinoma tissues [101]. In addition, ten herbs of CHM correlated with favourable survival outcomes in metastatic breast cancer patients and the bioinformatic analyses suggest inhibition of HSP90, ERα, and TOP-II related pathway as a potential mechanism of their anticancer action [97]. The potential anticancer effects of the phytochemicals were also evaluated in breast cancer survivors. Apart from the actual therapy of breast cancer, the understanding of whether post-diagnosis lifestyle (characterized by high intake of fruit and vegetable, low energy-dense food, regular physical activity, and healthy body weight) also affects the outcome of breast cancer survivors in terms of favourable inflammatory, metabolic, hormonal, and DNA methylation changes decreasing the risk of recurrence. A short-term dietary intervention on nutrition education exerted efficacy in increasing vegetable and fruit intake in Hispanic breast cancer survivors and altered biomarkers of breast cancer recurrence risk—borderline significant increase in global DNA methylation [102].

Last but not least, Teixeira et al. (2017) evaluated the bioavailability and metabolism of ellagitannins and anthocyanins after the consumption of native Brazilian cherry–grumixama fruit by humans, followed by the analysis of its anticancer activity in vitro. Eventually, phenolic acids and urolithin conjugates were the primary metabolites circulating and excreted in the urine. The subjects were classified as high and low urinary metabolite excretors demonstrating interindividual variability in the metabolism of polyphenols. Also, these metabolites were effective in inhibiting cell proliferation and G2/M cell cycle arrest in breast cancer MDA-MB-231 cells in vitro [103].

Despite already published clinical trials, several studies registered at clinicaltrials.gov deals with the potent efficacy of phytochemicals in breast cancer, for example:

- The intervention of whole-food, plant-based nutrition in metastatic breast cancer patients (ClinicalTrials.gov Identifier: NCT03045289)
- The response of overweight postmenopausal women with breast cancer to exercise and a plant-based diet (ClinicalTrials.gov Identifier: NCT04298086)
- The impact of plant extracted natural compounds on breast cancer survival time and regression at stage IV (ClinicalTrials.gov Identifier: NCT00910884)

In summary, the above-discussed results of clinical studies or overview of registered clinical trials point to a poor clinical analysis of the potential effects of phytochemicals without the intervention of conventional therapeutics to treat breast cancer.

## Potential limitations of effective use of phytochemicals in clinical practice

Extensive metabolism of naturally occurring plant secondary metabolites is considered a limitation of their bioavailability and bioactivity in vivo. The metabolization of phytochemicals occurs in the small and large intestine, and in the liver through associated phase I and II metabolism. Moreover, the absorption, distribution, metabolism, and elimination of phytochemicals and thus the circulating concentrations, elimination, and tissue exposure to the phytochemical are affected by age, sex, genotype, habitual diet, prescribed medications, and the gut microbiome. Despite the role of the factors on the side of the individual, the metabolism of phytochemicals is also affected by the structural complexity of phytochemicals alone [12, 104]. However, despite the described low bioavailability and bioactivity of phytochemicals in organisms, preclinical in vivo studies demonstrate a potent anticancer efficacy of whole plant food in animal models [29, 35, 42, 43, 48, 105]. Furthermore, some metabolites exert a better physiological function when compared with their precursor [12, 106].

Phytochemicals are generally considered safe and well-tolerated but several minor to more severe side effects have been also observed, e.g. mild gastro-intestinal symptoms, haemolytic anaemia, increased risk of hepatotoxicity, or toxic flavonoid-drug interactions [12, 107, 108]. Therefore, the correct dosage of phytochemicals is essential, depending on various factors, including the individual’s genetic background under the principles of predictive, preventive, and personalized medicine.

## Conclusions and expert recommendations for primary, secondary, and tertiary care in overall breast cancer management

Nanotechnology represents nanometric (< 500 nm) delivery systems that can enhance the bio-accessibility and bioavailability of hydrophobic compounds and can be used as drug-delivery vehicles, especially in targeted and combination therapy [109, 110]. In humans, phytochemicals exert poor bioavailability and absorption, rapid clearance, resistance, and toxicity. The reduction of mentioned disadvantages by using its nanoparticles is in the infancy of clinical trials but represents a huge potential in future cancer strategies [111]. Small particle sizes and unique materials used in delivery systems increase the stability and resistance to enzymatic activity in the gastrointestinal tract [112].

The extensive preclinical research revealed that phytochemical nanoparticles effectively target drug delivery with less/no side effects [113–118]. On the other hand, there is still insufficient evidence in the clinical sphere. Food and Drug Administration (FDA) approved several liposomes and polymer-based therapeutic nanoparticles for clinical use. The chemotherapeutic nanoparticles such as Doxil® (liposome-encapsulated doxorubicin), Abraxane® (albumin-bound paclitaxel), and Oncaspar® (PEG-Asparaginase) have emerged on the pharmaceutical market to date [111]. In terms of phytochemical nanoparticles, in a randomized, multicentre, parallel design in six female patients with invasive breast carcinoma (stage IIIA or IIIB), the nano-Ayurvedic medicine-gold nanoparticles-based drug along with the standard of care treatment (doxorubicin and cyclophosphamide) exhibited 100% clinical benefits compared to patients with standard of care only [118].

Nanomedicine, the alternative drug delivery and improvement of the treatment efficacy, could represent an innovative way in cancer treatment management in the future (e.g. replacement of current anticancer drugs). Still more detailed clinical studies are needed.

### Primary care is at the forefront of the paradigm change in breast cancer management from reactive to the predictive, preventive, and personalized approach

Breast cancer is a systemic multi-factorial disease initiated towards increasing health risks such as internal and external stress factors on one hand and, on the other hand, insufficiently effective protection mechanisms against the disease development [119]. The disease is progressing over years from sub-optimal health conditions to the clinical manifestation of potentially metastatic breast cancer. The time period between a reversible damage to health and irreversible organ damage with cascading complications is the operating timeframe for the primary care in breast cancer prediction and individualized protection. To this end, the majority of breast cancer cases are preventable that makes primary care to the key point in the paradigm change from the currently applied reactive disease management to the cost-effective 3PM approach [6, 7]. Health risk assessment followed by targeted damage-mitigating measures and protection tailored to the individualized patient profile are instrumental to reverse health damage at the level of sub-optimal health [120, 121]. As demonstrated above, an application of phytochemicals in the framework of primary care might be a particularly cost-effective approach exemplified by the evidence-based protection against the non-physiologic inflammation [35] and mitochondrial dysfunction [122] as well as against the Warburg malignant transformation [38]. In 2018, Polivka J Jr. et al. published a strategic article which later on has been distinguished by Springer-Nature *as groundbreaking scientific findings that could help humanity and protect our planet* in category “Medicine and Public Health”. The article presented new concepts of 3P medicine focused on the primary care and targeted prevention of one of the most aggressive and devastating BC subtypes, namely the pregnancy-associated breast cancer (PABC) [5]. The current article follows the principles and innovation of the distinguished publication and details a protective approach applicable to persons at high PABC risk.

### Secondary care: risk assessment, patient stratification, and targeted prevention of metastatic breast cancer

Secondary care in breast cancer management is essential to identify affected individuals and to protect them against breast cancer-associated metastatic disease. Early BC diagnosis utilising non-invasive liquid biopsy approach is highly beneficial to improve individual outcomes [8, 123]. Furthermore, from the predictive diagnostic point of view, specifically patients’ phenotyping, for example, characteristic for “pre-metastatic niches” in individuals with the Flammer syndrome phenotype [124] and liquid biopsy-based risk assessment are instrumental to estimate metastatic potential of clinically manifested breast cancer in each individual case. Multiomic targets including disease- and stage-specific cell-free nucleic acid patterns and enumeration of circulating tumour cells (CTCs density in blood stream) are highly recommended for secondary prevention in overall breast cancer management [8]. For the stratified patients, flavonoids per evidence are of great clinical utility as an effective sensitizer for anti-cancer therapy [125]. Finally, patients stratified as being highly predisposed to metastatic BC might strongly benefit from the administration of low-toxicity natural anticancer agents targeting specifically CTCs [126].

### Natural substances in secondary cancer care: potential protection of treated cancer patients against stroke

Treated cancer patients frequently die of a non-cancer cause well exemplified by stroke. Stroke in cancer may occur as a severe comorbidity or resulting from the cancer treatment; the described cases are non-bacterial thrombotic endocarditis, hypercoagulability, therapy, or direct tumour compression of blood vessels [127]. Bang et al. stated that one in seven to eight patients with ischemic stroke also has hidden or known cancer while cancer-related coagulopathy is a mechanism of stroke in 40% of them. Thrombin generation causing stroke related to coagulopathy is enhanced by both malignancy itself and cancer treatment (chemotherapy). Furthermore, a significantly increased risk of stroke may result from accelerated atherosclerosis—the side effect of radiotherapy. To this end, cancer and stroke share common risks such as vascular dysregulation and ischemic lesions [, 5, 124, 128, 129], further pronounced under the cancer treatment conditions [129, 130]. The study by Zaorsky et al. concluded that the risk of stroke among cancer patients is twice as high as that of the general population, particularly increasing later on in life. The study demonstrates high plurality of strokes in patients older than 40 years diagnosed with cancer of prostate, breast, and colorectum. Further breast cancer patients co-diagnosed with sleep disorders are at significantly increased stroke risk [131]. Consequently, risk assessment and advanced protective strategies in cancer treatments focused on individualized protection against stroke are strongly recommended [127].

Except cancer, naturally occurring phytochemicals and plant-based diet (rich in vegetable, fruit, grains, and olive oil also known as a Mediterranean diet pattern) exert efficacy in the protection against stroke, among others [132, 133]. The stroke-preventive activity of polyphenols is based on the effects on the cardio- and cerebrovascular system while their efficacy is suggested also when administered after the stroke onset and thus promoting the recovery of stroke patients [134]. An imbalance between antioxidants and pro-oxidants can result in cell damage, autophagy, apoptosis, and necrosis during stroke. To this end, potent anti-oxidative effects against stress protecting cells from damage are attributed to propolis that is rich in phenolics, flavonoids, and terpenes. Therefore, stroke-predisposed patients may strongly benefit from the daily supplementation with propolis [135]. Moreover, findings of the recently performed clinical study demonstrated that moderate habitual intake of flavonoid-rich foods results in lower risk of ischemic stroke [136]. Similarly, higher consumption of flavonoid-rich food (particularly citrus fruit, grapes, strawberries) was associated with a decreased risk of stroke in Japanese women [137].

The above-mentioned clinical trials demonstrating the efficacy of phytochemical-rich food in decreasing the risk of stroke have been conducted on subjects without cancer history [136, 137]. Further recent evidence supports biological effects of naturally occurring phytochemicals of potential clinical utility also against the cancer-related stroke. The flavonoid isoquercetin has been demonstrated to target protein disulfide isomerase (required for thrombus formation) and thus to improve coagulation markers in advanced cancer patients [138]. Based on the above discussed protective effects against cardiovascular and cerebrovascular damage among others, here we conclude a potential efficacy of naturally occurring phytochemicals against stroke as a frequent complications and cause of death in the patient cohort with treated cancer. Follow-up research to the topic is essential to advance secondary care in overall breast cancer management.

### Tertiary care of breast cancer: making palliation to the management of chronic disease

The motivation of the tertiary care is to advance palliation to the management of chronic disease. The task is highly ambitious, since breast cancer is known as capable to spread metastasis to the lymph nodes, bones, lung, liver, and brain, which are the most frequently reported sites of metastatic disease in BC [124]. Flavonoids and their nanotechnologically created derivatives have been reported as being of potentially great clinical utility for tertiary care. Their evident immune-supportive and drug-sensitizing effects which significantly increase sensitivity of malignant cells to anti-cancer therapies up to reversing anti-cancer therapy resistance, are a promising approach complementary to the therapeutic modalities currently applied within the tertiary care [125].

### Anti-COVID-19 protection of infected cancer patients

Several studies demonstrated that cancer patients including breast malignancies are at higher risk of severe illness and related deaths from COVID-19 infection. Managing cancer care under pandemic conditions is challenging. A multidisciplinary approach for optimal care of cancer patients in hospital settings is essential [139]. Due to their evident anti-inflammatory, anti-bacterial, and anti-viral properties, flavonoids are of great clinical utility in secondary and tertiary care of COVID-19-infected cancer patients including breast malignancies [125].

## Data Availability

Not applicable.
